# Design and test of potato seeding apparatus based on double-layer seed picking scoop structure

**DOI:** 10.1371/journal.pone.0295022

**Published:** 2023-12-21

**Authors:** Zhaomei Qiu, Yu Fang, Xin Jin, Jiangtao Ji, Xingyang Li, Yuxing Li

**Affiliations:** 1 College of Agricultural Equipment Engineering, Henan University of Science and Technology, Luoyang, Henan, China; 2 Longmen Laboratory, Science & Technology Innovation Center for Completed Set Equipment, Luoyang, China; Government College University Faisalabad, PAKISTAN

## Abstract

At present, potato seeders in China generally have poor uniformity of seed rows and high coefficients of variation in plant spacing during seed rows, causing difficulties for subsequent mechanized plant protection and harvesting. Based on the effect of seed discharge to analyze the sowing process, a potato seed discharger with a double-layer seed picking spoon structure was designed. By analyzing the seed discharging mechanism and its operation process, the shape and size structural parameters of the seed picking spoon were determined. Finite element simulation of the seed pickup process and seed carrying process of the seed discharging mechanism was carried out by EDEM software to determine the double-layer seed scoop scheme and the range of factors for subsequent tests. A two-factor test was conducted with seeding line speed and seed drop height as test factors, and plant spacing coefficient of variation and seed potato lateral offset dispersion as test indexes. The test results showed that the double-layer seeding spoon chain seeder reduced the coefficient of variation in plant spacing by 5.8%, and the dispersion in lateral offset by 5.5 mm, compared with the single seeding spoon seeder, when the seeding speed was 0.184 m/s and the height of falling seed was 9 cm.

## 1. Introduction

China’s potato production capacity has jumped to the first place in the world, and for many years China’s total planted area and total output of potato have been higher than those of other large producing countries [1, 2]. According to the National Bureau of Statistics, China’s potato cultivation area is 4,558 khm^2^ as of 2022. China’s potato unit yield is still lower than the global average, and less than half of the developed countries’ potato unit yields [3, 4]. The development of the potato industry is constrained by the low level of planting mechanization, a factor that has always been considered one of the most important factors hindering the development of the potato industry [5].

The full implementation of potato mechanization has now become the mainstream of the future development trend, sowing for the whole cycle of potato mechanized production of an important part. The quality of its planting operation has an important impact on the future harvest and product quality [6]. Currently, potato planters are used worldwide in a variety of forms, including many major types such as scooping scoops, turntables, conveyor belts, pins and needles, finger grips, and pneumatic types [7, 8]. The 9560 planter developed by Double L in the United States has the capability of continuous multi-row seeding, which is suitable for large field operations and improves operational efficiency [9]. The GL420 potato planter from Grimme, Germany, has a modular design and can plant four rows of potatoes at the same time [10, 11]. These large planters are suitable for application in Europe and the United States potato planting area is large, need large-scale planting area, to meet the needs of large-scale planting.

As the geographical environment of the main potato sowing areas in China is relatively harsh, mostly hilly and mountainous, it is not suitable for heavy machinery operation and the potato sowing precision requirements are high [12]. Wang et al. [13] designed an air-absorbing disc seed discharger for miniature potatoes based on the principles of vibratory seed supply and negative pressure suction. Lv et al. [14] designed an integrated blower for air-aspirated potato seeders, which employs an integrated blower to provide both negative pressure during seed pickup and positive pressure during seed delivery, realizing dual action of blowing and suction. The air suction seed discharge device has high requirements in terms of air tightness, seed potato size and power requirements. Wang et al. [15] investigated a finger-clamp potato planter, which utilizes a controlled opening and closing and swinging action of the clamp plate to pick up a single seed potato for seed placement. The pin-prick and finger-clamp types use a single grain to pick up the seed, which results in higher seeding accuracy, but also in severe damage to the seed potato and reduced speed of the seeding operation. Yang et al. [16] designed a seeder for potato breeding trials using a round-table lattice-disk seeding device, which allows seed potato seeding operations to be performed in the same position and with a more uniform spacing layout, but requires constant manual seed supply. Liu et al. [17] designed a vibratory sorting-based micro potato seeder, which utilizes the principle of forced vibration to achieve single-row sorting, conveying, and seed return operations for micro potatoes. The vibratory seed return process was analyzed to meet the seeding requirements for the national industry standard. At present, most potato planters use spoon chain seed discharge [18–21], which is simple in structure and widely applicable, but still suffers from a certain blindness in the process of active extraction of seed potatoes by the seed picking spoon [22]. The spoon seed discharge device commonly used on potato planters suffers from poor seed discharge uniformity [23]. And there is a problem of high coefficient of variation of plant spacing and lateral offset coefficient of seed potato planting due to the large uncertainty of the position of the seed potato thrown onto the back of the spoon in the seed guiding area. Therefore, in view of the above problems, this paper designs and develops a double-layer seed picking spoon chain potato seeder.

This paper develops a spoon chain seed discharge device for double-layer seed picking spoon structure, and designs key components such as seed discharge mechanism and seed picking spoon structure. By analyzing the seed potato seeding process, the test was conducted with the dual factors of seeding line speed and drop height as test factors, and the coefficient of variation of plant spacing and the dispersion of seed potato lateral offset as test indexes. The test showed that the double-layer seed picking spoon seeder can effectively improve the quality of potato sowing operation, and reduce the coefficient of variation of plant spacing and the dispersion of lateral offset of seed potatoes.

## 2. The machine structure and working principle

### 2.1 The machine structure

**Fig 1:**
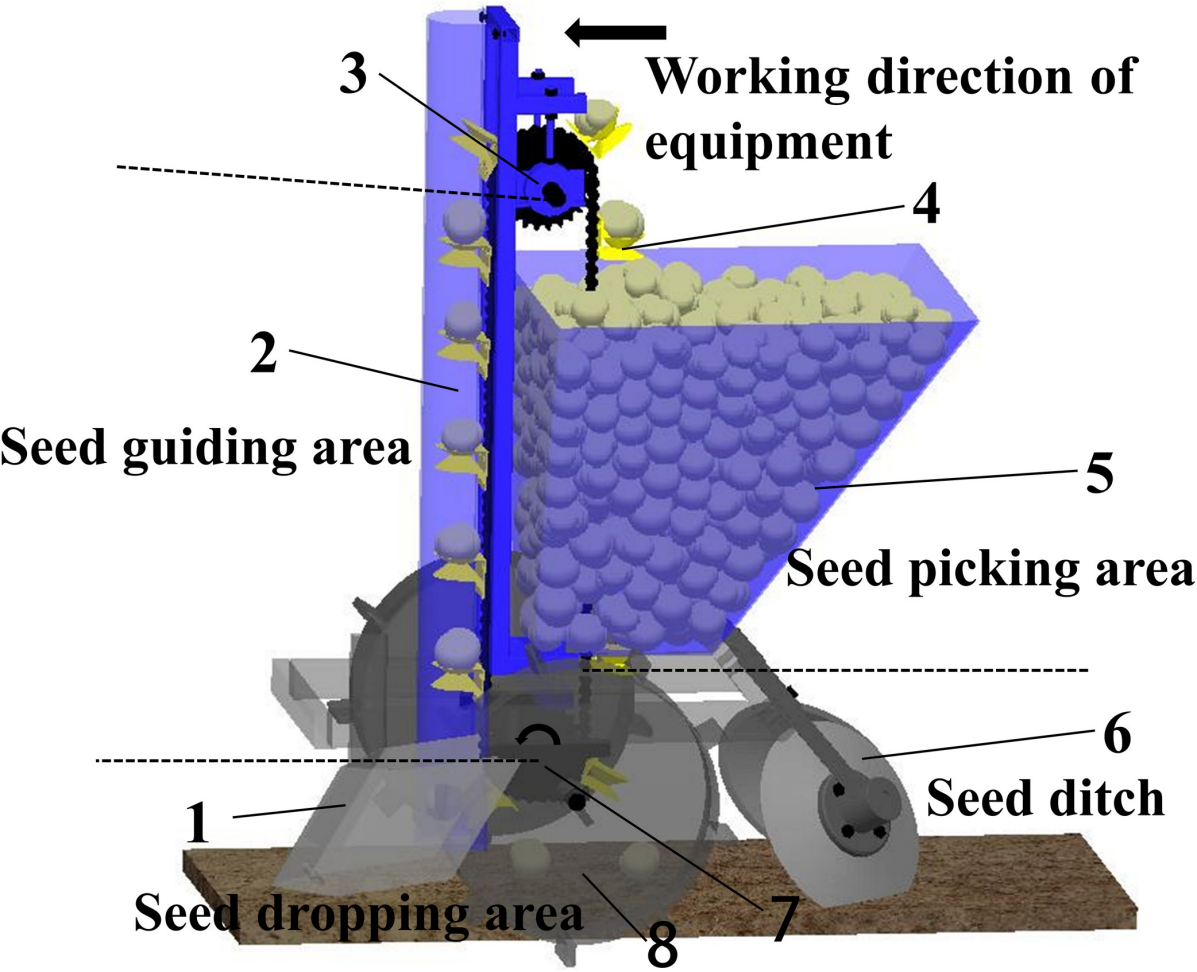
Schematic diagram of potato seed discharger mechanism based on double-layer seed picking spoon structure. 1. furrow opener, 2. seed guide shield, 3. driven sprocket, 4. double-layer seed picking spoon, 5. seed box, 6. mulching disk, 7. active sprocket, 8. ground wheel.

A potato planter is composed of several components such as a furrow opener, a seed discharge device, a ground wheel and a mulching device, as shown in Fig 1. The core component of a potato planter is the seed dispenser [24], the performance of which directly determines the quality of seeding. In this paper, the design of spoon chain potato seed discharger has been carried out. In order to reduce the coefficient of variation of plant spacing and the dispersion of seed potato lateral offset, a double-layer seed picking spoon structure was designed. According to the agronomic requirements of potato sowing operation and the shape distribution relationship of seed potato, at the same time to meet the sowing plant spacing in 16–30 cm index requirements.

### 2.2 Working principle

In the seeding operation, the seeder is towed forward by the tractor, and the ground wheel axle drives the seed discharge sprocket to rotate. The movement area of the seed potato seeding process is divided into three processes: seed picking area, seed guiding area and seed dropping area. In the seed picking process, the seed picking spoon moves from bottom to top inside the seed box, and the seed potatoes will be filled into the picking spoon, the size of the picking spoon only allows to pick up one seed potato, and the extra seed potatoes will be squeezed out without enough support. The seed picking spoon that obtains the seed potato continues to move, and when the seed discharger turns over the driven sprocket, the seed potato falls along the trajectory to the back seed spoon by the combined force. When the back of the seeding spoon turned over the sowing mouth, the seed potato loses its supporting force, and relies on the combined force it receives to drop down to the groove opened by the groove opener, completing the sowing process.

### 2.3 Agronomic requirements for potato sowing

**Fig 2:**
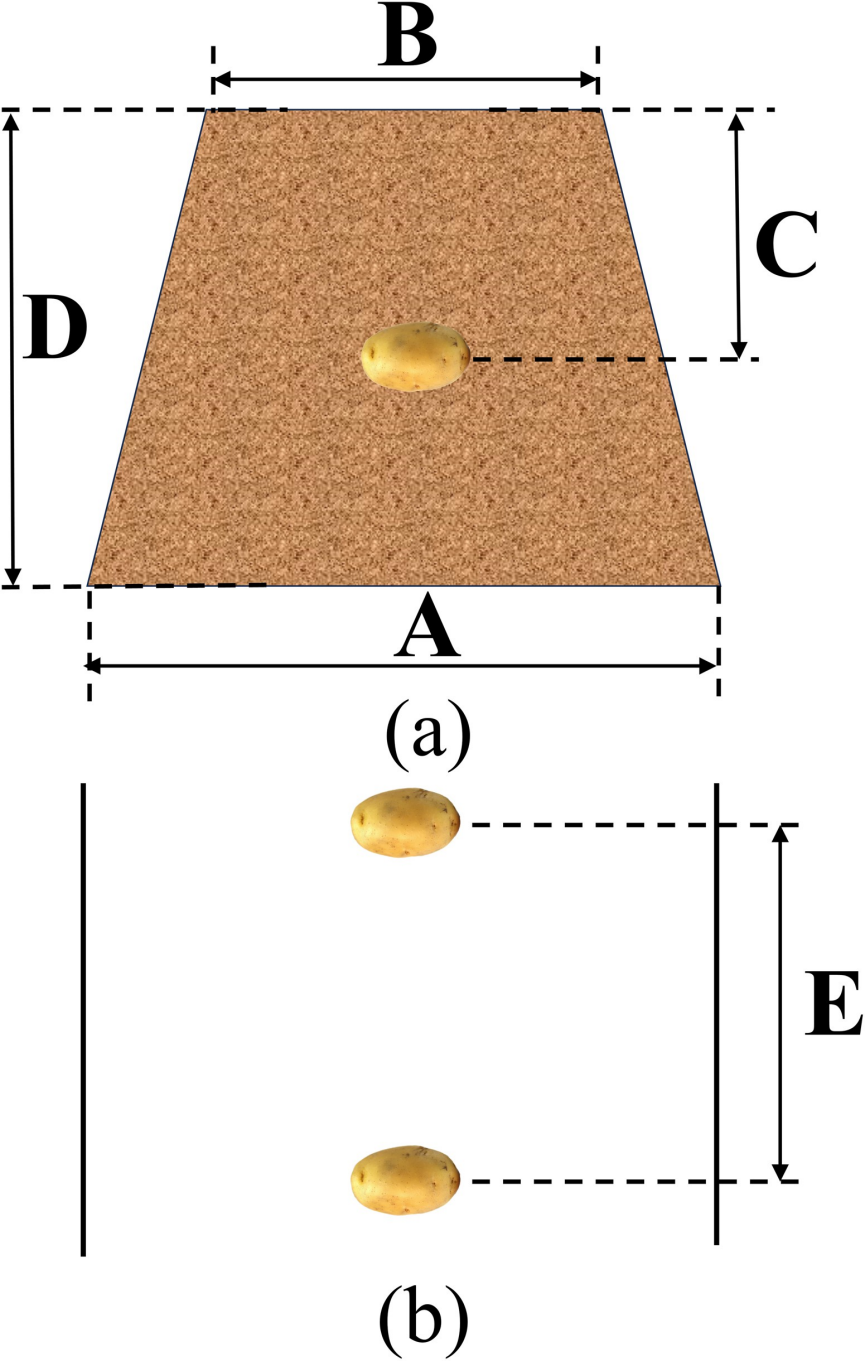
Agronomic requirements for potato sowing. (a). Front view of seed potato ridge. A. trapezoidal bottom width, B. trapezoidal top width, C. sowing depth, D. height of ridge. (b). Top view of seed potato ridge. E. plant spacing.

Potato planter planting is suitable for monocultures and single rows, and its agronomic requirements are shown in Fig 2. Where the lower bottom A of the trapezoidal ridge is between 60–90 cm, the upper top B of the trapezoidal ridge is between 40–60 cm wide, the sowing depth C is between 10–20 cm, and the height of the ridge D is between 20–25 cm, the plant spacing E is between 16–30 cm.

### 2.4 Seed potato physical research

The shape and size of the seed potato is directly related to the structural design and optimization of the dimensions of the pick-up scoop, pick-up area and seed guide area. In order to better understand the distribution of seed potato size and shape, to obtain more accurate information about seed potatoes, and to design the seed discharge device more rationally. In this paper, 100 small seed potatoes were randomly selected from the seed potato bank, and the three-axis dimensions of length, width and thickness of the seed potatoes were measured with electronic vernier calipers to obtain the approximate distribution of seed potato dimensions. The seed potato shape *f* index is used to define the shape characteristics of seed potatoes. The statistical results are shown in Tables 1 and 2.

**Table 1:** Index of the potato shapes.

Potato shape	Index
**Round**	100–160
**Oval**	≥161–240
**Long-stripped**	≥241–340
**Extra-long stripped**	>340

**Table 2:** Average index of size and shape of small seed potatoes.

Length (mm)	Width (mm)	Thickness (mm)	Three-axis mean size (mm)	Shape index
54.23	43.52	37.36	45.04±6.97	186.64

The shape index is calculated as [25]:

$$f=\frac{L^{2}}{W\times t}\times 100$$ (1)

Where L is the maximum length of the seed potato (mm); W is the maximum width of the seed potato (mm); t is the maximum thickness of the seed potato (mm).

**Fig 3:**
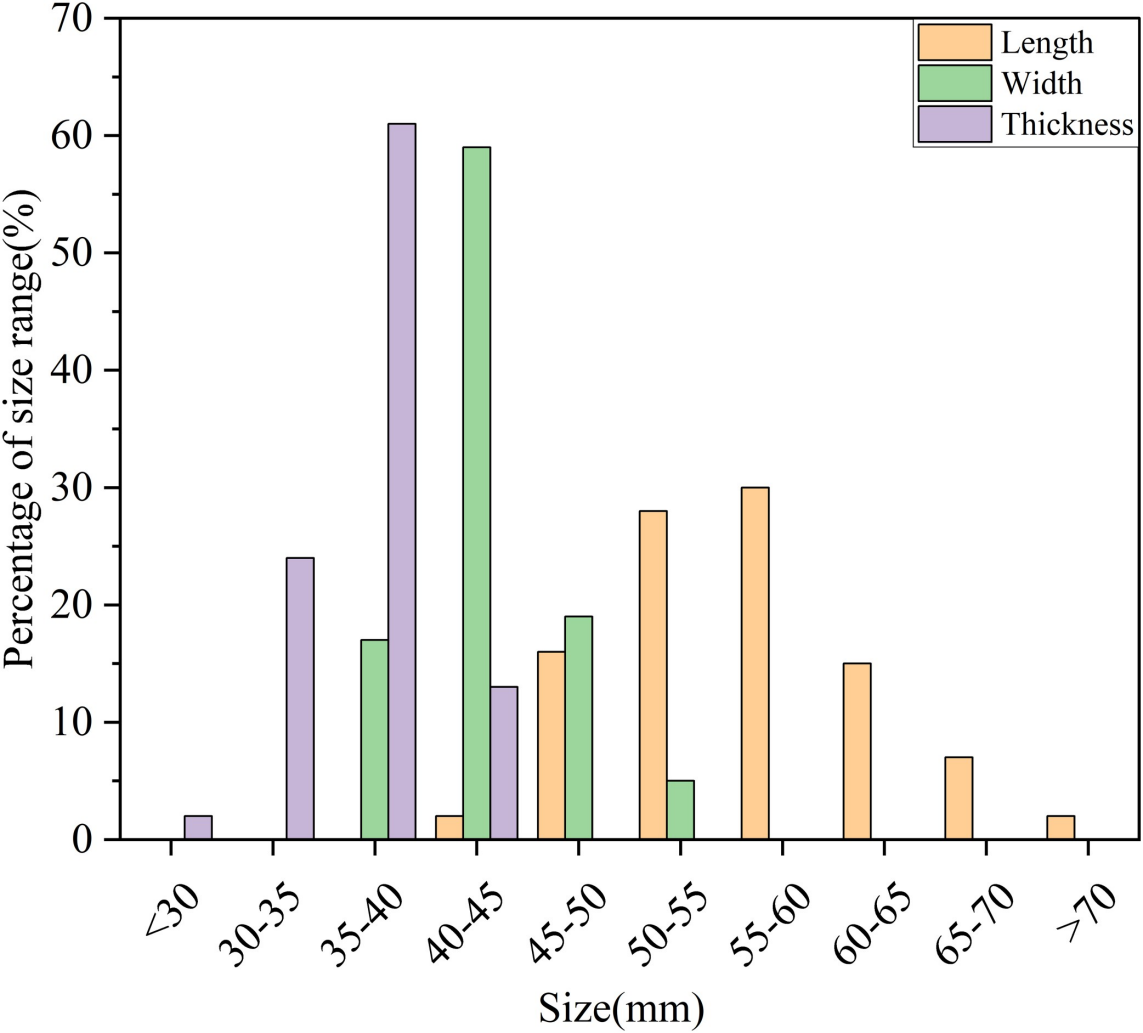
Triaxial size distribution of seed potatoes.

According to Tables 1 and 2, it can be seen that most of the small seed potatoes were oval in shape. From the analysis of Fig 3, it can be concluded that the length, width and thickness of seed potatoes were concentrated between 50–60 mm, 40–45 mm and 35–40 mm respectively.

### 3. The key component design

#### 3.1 Design of seed spoon dimensions

**Fig 4:**
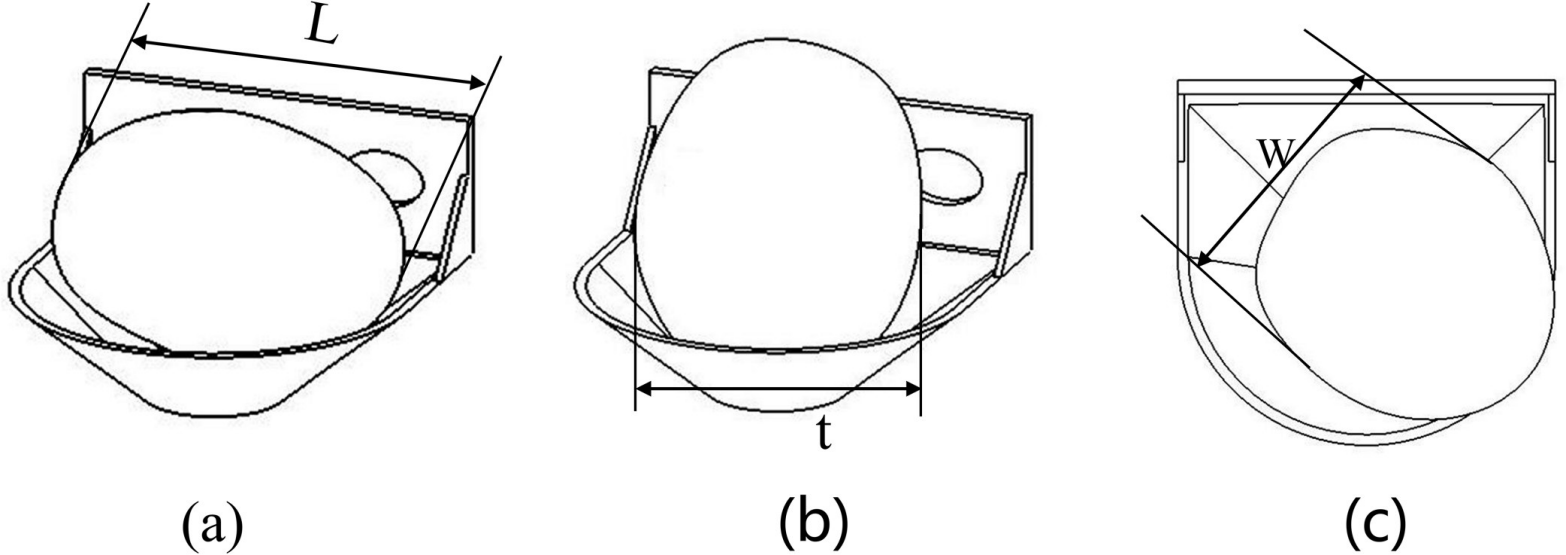
Seed potato postures. a. Seed potato lying on its side, b. Seed potato lying flat, c. Seed potato in upright position.

The design of the seed picking spoon has a crucial impact on the accuracy and success rate of seed picking, in order to successfully complete the seed picking process, the design of the seed picking spoon should meet the following requirements: (1) avoid the overlap of two smaller seed potatoes on the same seeding spoon; (2) the largest size of the selected seed potatoes can have a large area of contact with the seeding spoon. The attachment state of seed potato in the seed picking spoon can be in 3 positions: side-lying, flat-lying and upright. This is shown in Fig 4 below. In the seed box seed picking process, the seed potato will be pushed to the most stable state because the seed potato will be affected by the synergistic force between the seed potatoes.

**Fig 5:**
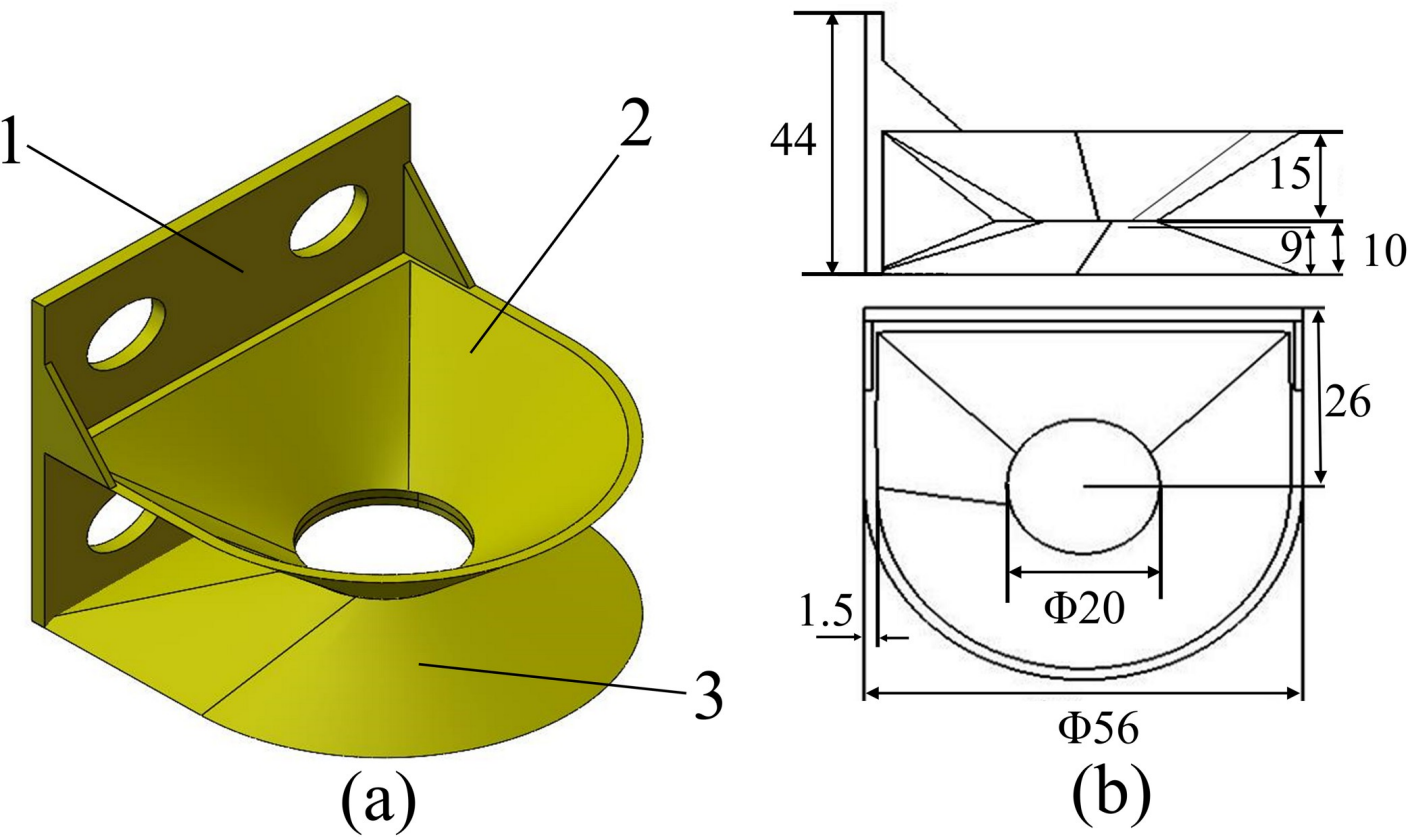
Dimensional drawing of the structure of double-layer seed picking spoon. (a) Structure of double-layer seed picking spoon: 1. spoon handle, 2. frontal seed pick-up spoon, 3. dorsal seed pick-up spoon. (b) Dimensional drawing of the seed scoop.

The design of the seed picking spoon needs to comply with the requirements of the parameter range of seed potato sizes, so that after the seed picking process is completed, the seed potatoes fall to the backside without a fixed position when the seed is thrown. In order to be able to make the spacing change of the seed potatoes as small as possible as they fall onto the back seed scoop. In order to make the plant spacing change of seed potatoes falling onto the back seed spoon as small as possible, a double-layer seed picking spoon structure is designed. One frontal seed scoop is used during the seed pickup process, while the other back seed scoop receives the seed potatoes tipped off by the next seed scoop. The structural dimensional parameters of the seed picking scoop depend mainly on the shape of the seed potato. In general, the maximum diameter D of the seed spoon surface and the depth H of the seed spoon should satisfy [26]: $\text{D}>\bar{\text{L}}>\bar{\text{t}}>2\text{H}$. The diameter D of the seed scoop is greater than the average length $\bar{\text{L}}$ of the seed potato, such that the seed potato can be surrounded by the seed scoop. The average length $\bar{\text{L}}$ of the seed potato is greater than the average thickness $\bar{\text{t}}$. The designed frontal seed pickup scoop depth H should be less than half of the average thickness $\bar{\text{t}}$ of the seed potato, because the deeper the seed potato sinks into the frontal seed pickup scoop causes an increase in the contact area of the other seed potato with the spoon handle 1, which increases the reseeding rate. It was determined that the triaxial dimensions of the designed seed spoon were 28 mm maximum radius, 15 mm depth of the spoon mouth, and 26 mm distance from the center to the spoon handle, when the seed pickup rate was high. The structural dimensions of the seed spoon are shown in Fig 5.

### 3.2 Seeding chain design

According to the agronomic characteristics of monoculture single-row potato planting technology, *Z*_1_ = 20 and *Z*_2_ = 20 were selected through the handbook of machine design [27]. The transmission ratio *i* is:

$$i=\frac{Z_{2}}{Z_{1}}=\frac{20}{20}=1$$ (2)

Where *Z*_1_ represents the number of teeth of the active sprocket and *Z*_2_ represents the number of teeth of the driven sprocket.

Chain center distance should not be too small, at the same time the chain and sprocket meshing more times will lead to their increased wear. In addition, the distance between the center of the chain and the sprocket should not be too large, otherwise the vibration amplitude increases when the seed picking spoon transports seed potatoes. From the design manual, the chain drive center distance a is usually taken as 30-50*p*, and the initial selection *a* = 32 *p* = 540. The number of chain links is: The number of chain links *L*_*p*_ is even, calculated as in Eq (3), so *L*_*p*_ is taken as 84.

$$L_{p}=2\times \frac{a}{p}+\frac{Z_{1}+Z_{2}}{2}+{\left(\frac{Z_{2}-Z_{1}}{2\pi }\right)}^{2}\times \frac{p}{a}$$ (3)

Where the center distance of the chain drive is a, the pitch is *p*, the number of teeth of the active sprocket and the driven sprocket are *Z*_1_ and *Z*_2_, and the number of chain links is *L*_*p*_.

Chain length is:

$$L=L_{p}\times p$$ (4)

The maximum center distance of the chain drive is:

$$a=\frac{p}{2}\;\left(L_{p}-Z_{1}\right)$$ (5)

The design of the spoon chain seed discharger should be evenly distributed on the entire seed discharge chain 14 seed scoop, from the above calculations: seed chain model selection of 10A sleeve roller chain, pitch *p* = 15.875 mm, the number of chain links *L*_*p*_ = 84, then the seed scoop spacing:

$$l=L_{p}\times p\;÷14=84\times 15.875÷14=95.25mm$$ (6)

The seed pickup speed of the discharge chain has a large impact on the seeding quality, and the seed pickup line speed generally should not exceed 0.5 m/s [28]. When the sprocket rotational speed is *n*_1_, the chain takes the seed linear speed *v* as:

$$v=\frac{2\pi n_{1}}{60}\times r$$ (7)

The linear speed of the seed discharge chain and the linear speed of the seed bed belt are determined by the plant spacing *L*, and the time it takes for the seed bed belt to move through one plant spacing is equal to the time it takes for the seed discharge chain to move through one seed scoop spacing *l* [29]. Let the linear velocity of the seed discharge chain be *v* and the linear velocity of the seed bed belt be *v*′, then:

$$\frac{L}{v^{\prime }}=\frac{l}{v}$$ (8)

According to the agronomic requirements plant spacing is set to 20 cm, the speed of spoon chain seed extraction is taken as 0.1–0.4 m/s, and the speed of seed bed belt is 0.21–0.84 m/s.

### 3.3 Analysis of the movement process of the seed discharger

**Fig 6:**
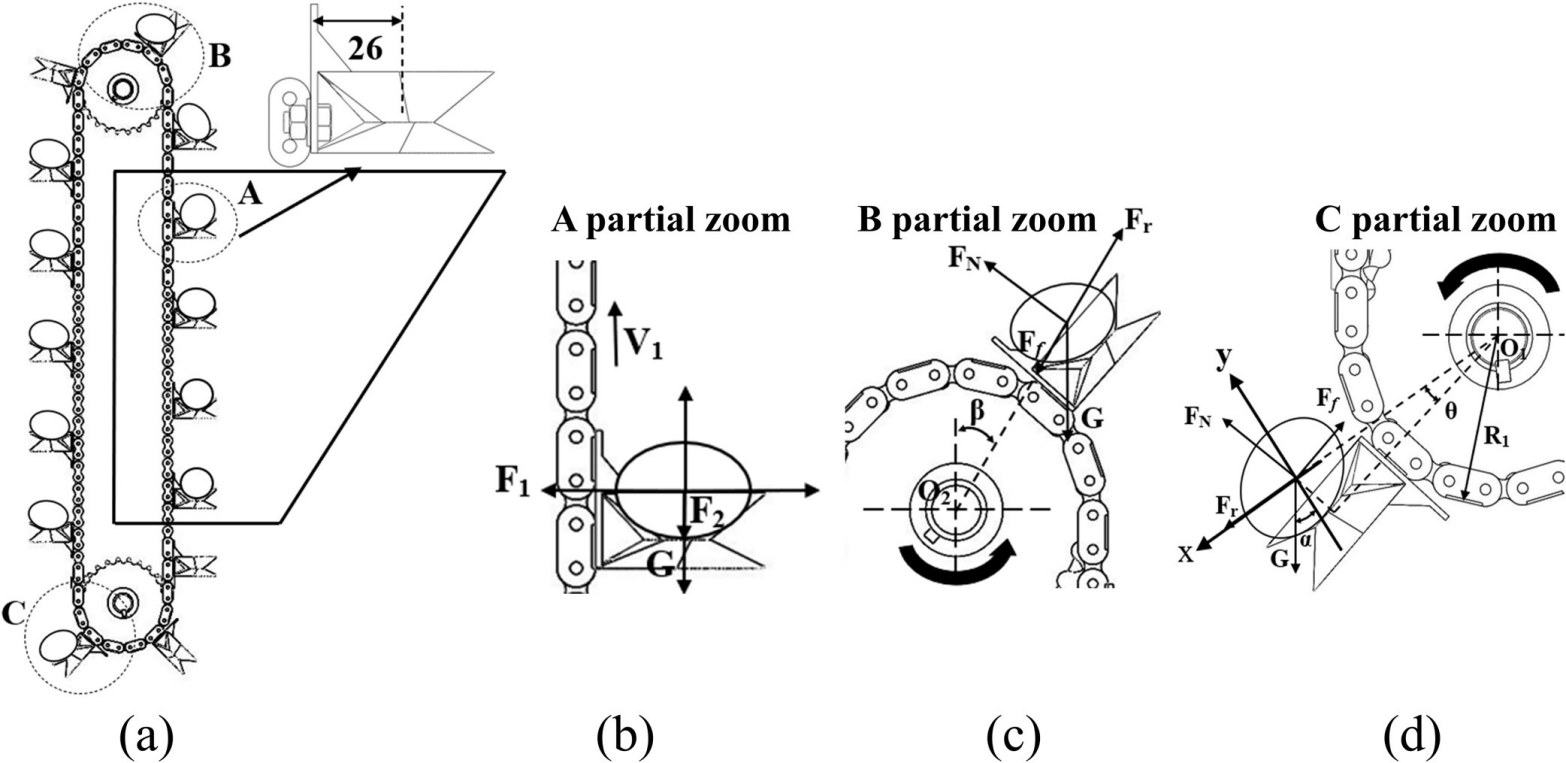
Seed potato movement process and force analysis. a. Seed potato filling and discharging movement processes, b. Force diagram of seed potato filling process, c. Seed potato feeding force diagram, d. Seed potato initial drop force diagram.

Seed potatoes are put into the ridge from the seed box through the seed picking area, seed guiding area and seed dropping area, and the seed picking and seed guiding process is shown in Fig 6(a). In the seed picking area, the seed picking spoon picks up seeds sequentially in the seed box with the movement of the chain. In this process, the seed potato force is balanced, as shown in Fig 6(b), and the seed potato force satisfies the following relationship:

$$\left\{\begin{array}{c} {\text{F}}_{N}={\text{F}}_{2}+G\\ {\text{F}}_{f}={\text{F}}_{1} \end{array}\right.$$ (9)

Where F_*f*_ is the friction between seed potato and seed picking spoon, N; F_1_ is the transverse synergistic pressure between seed potatoes, N; F_2_ is the longitudinal synergistic pressure between seed potatoes, N; and *G* is the gravitational force of seed potato, N.

Seed potato will be thrown to the back of the previous seed picking spoon when it reaches the region of the driven sprocket, and the force diagram is shown in Fig 6(c), and the threshold condition that the seed potato maintains the relative equilibrium and is not thrown away should satisfy the following relationship:

$$\left\{\begin{array}{c} {\text{F}}_{N}=Gsin\beta \\ {\text{F}}_{f}+Gcos\beta \geq {\text{F}}_{r} \end{array}\right.$$ (10)

Where F_*r*_ is the inertia force, N; *β* is the rotation angle of the seed picking spoon, (°). In the seed throwing stage, the greater the speed, the greater the inertia force, the smaller the angle at which the seed picking spoon turns, the seed potato will be thrown out, deviating from the seed picking spoon trajectory.

At the initial seed drop point, with the seed potato center of mass as the origin, establish a right-angle coordinate system, the x direction is in the same direction as the inertia force, and the y direction is perpendicular to the inertia force, as shown in Fig 6(d), and the force on the seed potato satisfies the following relationship:

$$\left\{\begin{array}{c} {\text{F}}_{N}cos\theta +{\text{F}}_{f}sin\theta -Gcos\alpha =0\\ {\text{F}}_{N}sin\theta +{\text{F}}_{f}cos\theta -Gsin\alpha \leq {\text{F}}_{r} \end{array}\right.$$ (11)

Where *θ* is the residual angle of the angle between the inertia force F_*r*_ and the support force F_*N*_, (°); *α* is the angle between gravity G and the y-axis.

The inertia force and the friction between the seed potato and the picking spoon satisfy the following relationship:

$$\left\{\begin{array}{c} {\text{F}}_{r}=\frac{mv_{1}^{2}}{\sqrt{{\left(R_{1}+26\right)}^{2}+{\left(t/2\right)}^{2}}*10^{-3}}\\ \begin{array}{c} {\text{F}}_{f}=\mu {\text{F}}_{N}\\ G=mg\\ tan\theta =\frac{t/2}{R_{1}+26} \end{array} \end{array}\right.$$ (12)

Where *μ* is the friction coefficient of seed potato and seed picking spoon, and *μ* is taken as 0.445; *R*_1_ is the radius of sprocket wheel, mm; *t* is the thickness of seed potato, mm. Combining Eqs (11) and (12) yields the following relationship:

$$v\geq \sqrt{0.487cos\alpha -0.812sin\alpha }$$ (13)

When the linear velocity is certain, with the increase of angle *α*, the combined force of the seed picking spoon on the seed potato is smaller, and when it reaches a certain angle, the seed potato will fall. At the initial seed drop point, the greater the pickup line speed, the greater the inertia force, in the pickup spoon turned at a relatively small angle seed potato is thrown.

### 3.4 Simulation analysis of seeding process

**Fig 7:**
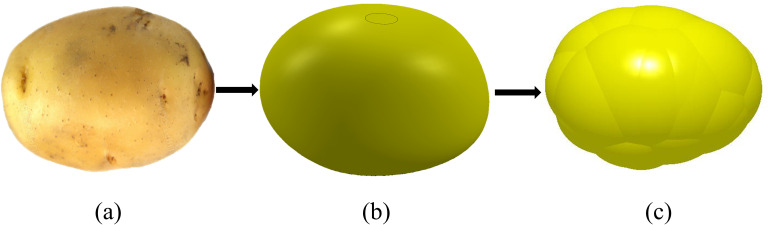
Potato pellet model. a. original model, b. SolidWorks modeling model, c. EDEM filled model.

In order to further study the movement process of seed potato in the seed discharger, this paper utilizes the discrete element software to carry out simulation tests on the seed discharger in the potato seed picking and seed dropping process. As shown in Fig 7, the length, width and thickness dimensions of the seed potatoes were measured manually using vernier calipers to obtain an average length of 51.8 mm, a width of 40.8 mm and a thickness of 35.6 mm for the seed potatoes. The average size of the measured potatoes was used to establish the seed potato contour model in SolidWorks software, and then the seed potato particle model was obtained by filling the seed potato model with a multi-faceted ball using EDEM software.

In order to facilitate the simulation and computation, the built model of the seed discharger was simplified by deleting the part that has no contact with the particles, and imported into EDEM to establish the motion parameters and contact parameters. The parameter settings [30, 31] used for the model in the simulation are shown in Table 3 below.

**Table 3:** EDEM simulation parameter settings.

Material or Contact	Property	Unit	Value	Source
**Potatoes**	Poisson’s ratio		0.57	Literature [30]
	Shear modulus	Pa	1.336×10^6^	Literature [30]
	Density	kg/m^3^	1048	Literature [30]
**Plastic**	Poisson’s ratio		0.35	EDEM Library
	Shear modulus	Pa	1.3×10^9^	EDEM Library
	Density	kg/m^3^	1200	EDEM Library
**Machine**	Poisson’s ratio		0.30	EDEM Library
	Shear modulus	Pa	7.0×10^10^	EDEM Library
	Density	kg/m^3^	7800	EDEM Library
**Potatoes—Potatoes**	Coefficient of restitution		0.79	Literature [31]
	Static-friction coefficient		0.452	Literature [30]
	Rolling-friction coefficient		0.024	Literature [30]
**Potatoes—plastic**	Coefficient of restitution		0.66	Literature [31]
	Static-friction coefficient		0.517	Literature [30]
	Rolling-friction coefficient		0.301	Literature [30]
**Potatoes—machine**	Coefficient of restitution		0.71	Literature [31]
	Static-friction coefficient		0.445	Literature [30]
	Rolling-friction coefficient		0.269	Literature [30]

**Fig 8:**
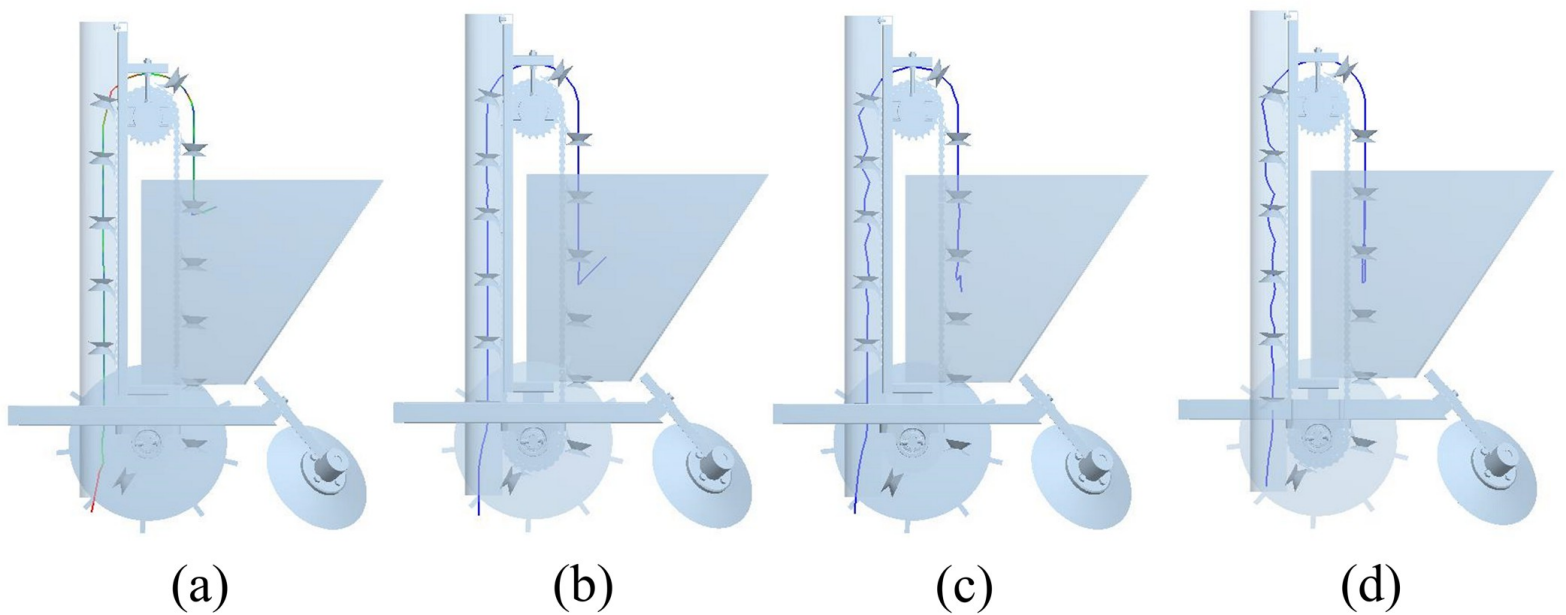
Seed potato trajectories. a. 0.1 m/s seed pick-up trajectory, b. 0.2 m/s seed pick-up trajectory, c. 0.3 m/s seed pick-up trajectory, d. 0.4 m/s seed pick-up trajectory.

**Fig 9:**
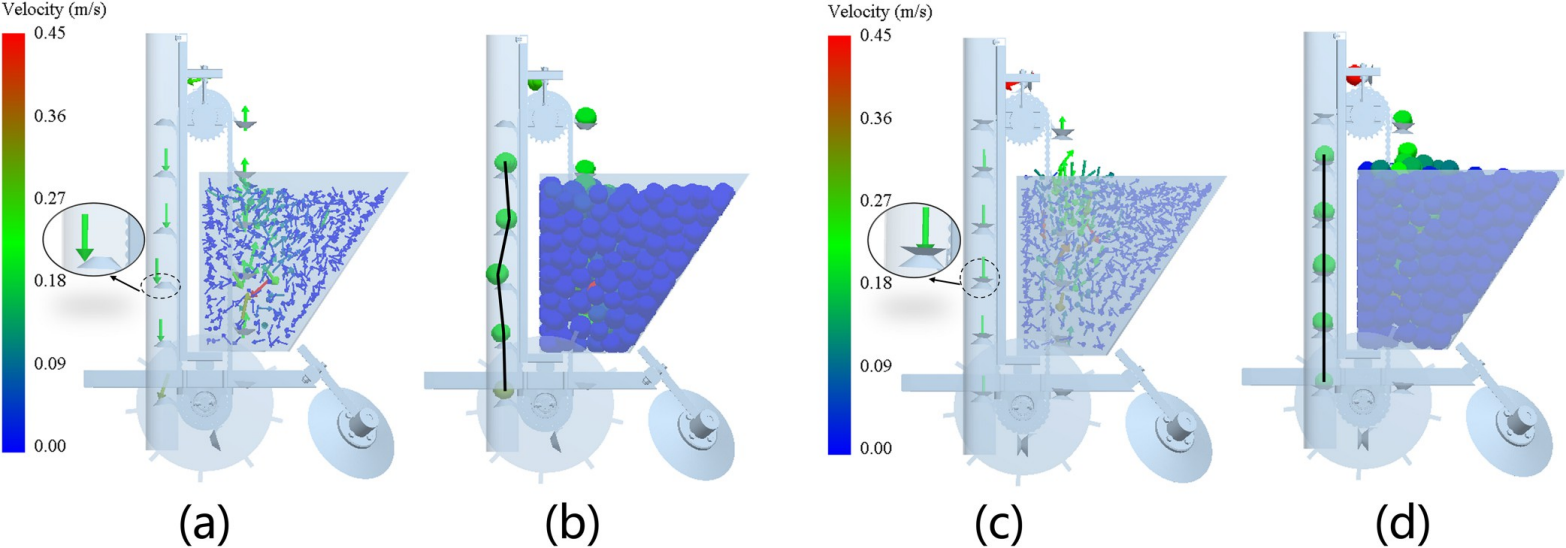
Simulation comparison test of single and double spoon seeding process. a. Velocity vector display of the seed discharge process with a single pick-up spoon, b. Seeding process for single pick-up scoop seed potato display, c. Double-layer seed scoop speed vector showing the seed discharge process, d. Seeding process with double pick-up spoon seed potato display.

In this simulation test, the seed discharge chain was used to pick up seeds at a linear velocity of 0.1–0.4 m/s. In the seed picking process, a seed potato in the discharge process of the trajectory process shown in Fig 8, the seed potato in the experience of seed picking area, seed guiding area and seed dropping area after falling into the groove, you can see that the seed potato is relatively smooth completion of the whole process from the seed box to the groove. In the simulation of Fig 8(a) and 8(b), it can be seen that the seed potato trajectory is more stable in the seed guiding area when the seed is taken at 0.1 m/s and 0.2 m/s speeds. While in Fig 8(c) and 8(d) simulation it can be seen that the seed potato trajectory fluctuates more at 0.3 m/s and 0.4 m/s as well as upward velocity. In the seed throwing stage, the seed potato was thrown when the angle of the seed picking spoon turned over was relatively small, verifying the results of the force analysis mentioned above. And from the seed throwing trajectory, the greater the speed will touch the seed guide tube, increasing the possibility of seed potato damage. Now choose 0.2 m/s speed for single and double seeding spoon comparison simulation test, seed potatoes are thrown to the back of the single seeding spoon, due to the inconsistency of the landing point, the plant spacing appeared to be a large offset; the design of the double-layer seeding spoon can reduce the seed potatoes in the sowing process of the plant spacing offset. The simulation comparison results are shown in Fig 9.

From the simulation comparison test, it can be seen that in the process of using a single spoon to pick up seeds and a double spoon to discharge seeds, the seed potatoes have better mobility in the seed box, and can be picked up smoothly in the seed picking area. We can see that (a) and (c) in Fig 9 are velocity vector diagrams of seed potato during seeding. By observing the velocity vector diagrams, we can understand the change of the size and direction of the velocity of the seed potato in the seeding process, and can also more intuitively reflect the flow of the seed potato in the seed box. In Fig 9(a) enlarged diagram, due to the seed potato velocity vector falling point deviation from the center of the seed spoon distance in the seed guide cover closer position, need to guide the seed cover and take the seed spoon under the joint action of the downward movement, increase can be on the design of the seed guide cover requirements. In Fig 9(c) a part of the partial enlargement of the picture, the seed potato velocity vector landing point in the center of the seed spoon, increasing the straightness in the seed guide area. And the seed potato particles displayed in Fig 9(a) and 9(c) correspond to Fig 9(b) and 9(d) respectively. It can be seen from Fig 9(b) and 9(d) that the double-layer seed picking spoon structure makes the seed potato have better stability and straightness in throwing and falling into the seed guiding area; the seed potato can fall in the same position on the backside of the seed spoon, which reduces the offset distance when it falls into the furrow.

## 4. Seeding performance test

### 4.1 Construction of the test bench

**Fig 10:**
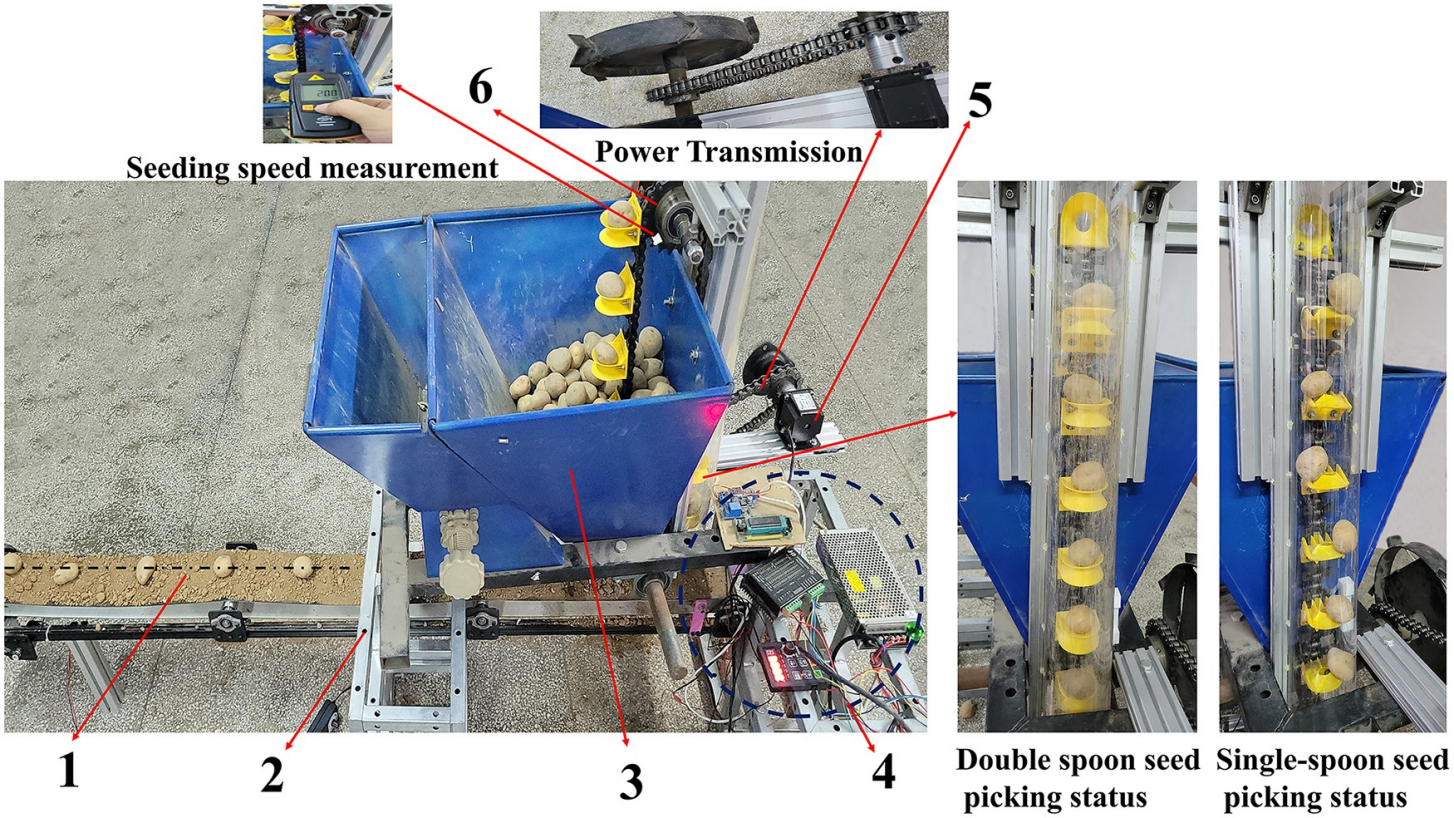
Structure of the test bench. 1. seed bed belt device, 2. Frame, 3. seed box, 4. control components, 5. stepping motor, 6. spoon chain seed discharger.

In order to comply with the design requirements, a potato sowing quality detection test was carried out in the laboratory. As shown in Fig 10, a test bench for potato sowing quality testing was designed to conduct the relevant tests.

The test bench is mainly composed of frame, stepping motor, seed bed belt device, control parts and spoon chain seed discharger. In the test process to simulate the planter field operation situation, that is, this seed discharger by the tractor traction forward operation. Now the position of the seed dispenser relative to the ground remains unchanged, the seed bed belt moves relative to the seed dispenser, and the seed extraction power of the seed dispenser is provided by a stepping motor. The stepper motor transmits power to the ground wheel through the sprocket and chain parts, and the ground wheel rotates to drive the active sprocket. Seed picking speed can be monitored by a tachometer and adjusted by a stepper motor controller. The conveyor belt moves in the opposite direction to the forward direction of the planter to simulate the forward movement of the planter, and the forward speed of the conveyor belt can be adjusted to simulate different planting environments to adapt to different planting speeds. Due to the coefficient of elasticity between the conveyor belt and the seed potatoes, the fall on the conveyor belt rolls and bounces, which adversely affects the measurement of the test results [32]. The use of artificial in the seed bed belt constantly spread 2 mm thick sandy loam, better simulation of seed potato fall to the ditch condition, can significantly reduce the seed potato landing point at the jump.

### 4.2 Test apparatus and materials

Seed potatoes were selected from Hubei Enshi Marco seed potatoes. The shape index was 185, the average moisture content was 77.3%, the average mass of single seed potato was 38.6 g, and the clarity was more than 99%. The instruments used in the test were straightedge, 5 m tape measure, electronic vernier caliper, and tachymeter.

### 4.3 Test indicators

**Fig 11:**
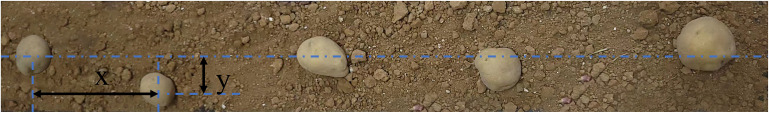
Schematic diagram of test indicators.

Referring to the test methods and indexes stipulated in GB/6242-2006 "Planting Machinery Potato Planter Test Methods" and NY/T1415-2007 "Technical Specification for Quality Evaluation of Potato Planters", test the operational performance of the double-layer seed picking spoon chain-type potato seed discharger. The coefficient of variation of plant spacing x and the dispersion of lateral offset of seed potatoes y were selected as evaluation indexes for this experiment, as shown in Fig 11. A two-factor combination test was conducted using seed discharge speed and seed drop height as test factors, and each set of tests was repeated three times to calculate the mean of the measurements.

Seed potato spacing offset dispersion is the offset of the distance x of seed potato projected on the axis from the average plant spacing. It is an important indicator for evaluating the longitudinal distribution of seed potatoes in the seed bed. A total of 100 seed potatoes were selected for plant spacing measurements during each sowing and the coefficient of variation for plant spacing was calculated as follows in Eqs 14 to 15.

$$\eta =\sqrt{\frac{1}{J}\overset{J}{\underset{i=1}{\sum }}{\left(x_{i}-\bar{x}\right)}^{2}}$$ (14)

$$\partial =\frac{\eta }{\bar{x}}\times 100\%$$ (15)

Where *η* is the seed potato spacing offset dispersion, mm; *x*_*i*_ is the actual spacing, mm; $\bar{x}$ is the average spacing, mm; and *J* indicates the number of experimental data measurements. *∂* is the coefficient of variation of spacing, %.

Seed potato lateral offset dispersion refers to the degree of seed potato deviation from the axial baseline, and is an important indicator for evaluating the lateral distribution of seed potato in the seed bed. Based on the midline of the furrow, the lateral offset dispersion of 100 seed potatoes in each measurement was calculated. The calculation equation is as follows [33]:

$$\bar{d}=\frac{\sum_{i=0}^{n}\left|y_{i}-\bar{y}\right|}{n}$$ (16)

Where $\bar{d}$ is the dispersion of seed potato lateral offset, mm; *y*_*i*_ is the value of seed potato lateral offset, mm; $\bar{y}$ is the mean value of seed potato lateral offset, mm; and *n* is the number of experimental data measurements.

### 4.4 Experimental program and analysis of results

#### 4.4.1 Experimental design

In order to clarify the influence of seeding speed and drop height on the uniformity of seed discharge, the seeding chain spoon line speed was selected 0.1 m/s, 0.2 m/ and 0.3 m/s as the test factor level. Seed drop height has a greater impact on the performance of seed dispenser uniformity, and the seed drop height is based on the distance between the seed guide shield and the ground [34]. Reference took 9 cm, 12 cm and 15 cm as the factor levels of seed drop height test [35]. During the course of the experiment, the speed of seed discharge and the seed drop height of the seed discharger can be adjusted according to the different field conditions in the simulation. After the test, the significance of the test indexes plant spacing coefficient of variation and seed potato lateral offset dispersion were analyzed, and the optimal combination of factor parameters was selected according to the actual situation. The coding table of the levels of the test factors is shown in Table 4. Experimental design and results are shown by the S1 Table in S1 File, and the corresponding test results were compared by Duncan’s method as shown in Table 5. The results of the between-subjects effects test are shown in Table 6.

**Table 4:** Test factors and levels.

Level	Experimental factors	
	Seeding speed A (m/s)	Seed drop height B (cm)
**+1**	0.3	15
**0**	0.2	12
**-1**	0.1	9

**Table 5:** Results and comparisons using Duncan.

	Coefficient of variation for plant spacing (%)			Seed potato lateral offset dispersion (mm)		
B	-1(9)	0(12)	1(15)	-1(9)	0(12)	1(15)
A						
**-1(0.1)**	14.37±0.90 a A	15.97±0.38 b B	17.13±0.67 b B	7.97±0.21 a A	8.57±0.35 a B	10.07±0.81 b A
**0(0.2)**	13.27±0.47 a A	12.9±0.72 a A	16.1±0.44 b A	7.53±0.35 a A	6.83±0.31 a A	9.1±0.62 b A
**+1(0.3)**	16.23±0.38 a B	17.87±0.25 b C	19.9±0.36 c C	9.5±0.3 a B	10.63±0.55 b C	12.5±0.2 c B

Note: Using Duncan post-hoc comparisons, tables containing different lowercase letters indicate significant differences (p<0.05) between values at different B-factor levels for the same A-factor level; Tables containing different capital letters indicate significant differences (p<0.05) between the values of different A factor levels for the same B factor level.

**Table 6:** Results of the between-subjects effects test.

Response	Variance Source	Sum of Squares	Degree of Freedom	Mean Square	F Value	P Value
**Coefficient of variation for plant spacing *Y***_**1**_ **/%**	Model	119.36	8	14.92	50.29	<0.0001**
	A	69.13	2	34.57	116.51	<0.0001**
	B	45.02	2	22.51	75.87	<0.0001**
	A×B	5.21	4	1.3	4.39	0.0119*
	Pure Error	5.34	18	0.2967		
	Cor Total	124.70	26			
**Seed potato lateral offset dispersion *Y***_**2**_ **/%**	Model	72.29	8	9.04	43.72	<0.0001**
	A	43.42	2	21.71	105.04	<0.0001**
	B	25.75	2	12.87	62.30	<0.0001**
	A×B	3.12	4	0.7806	3.78	0.0212*
	Pure Error	3.72	18	0.2067		
	Cor Total	76.01	26			

Note:

** indicates that the item is highly significant (P < 0.01);

* indicates that the item is significant (P < 0.05).

(1) Significance analysis of the coefficient of variation *Y*_1_ of the plant spacing

By analyzing the data, the results of the between-subjects effect test for the coefficient of variation of spacing *Y*_1_ is shown in Table 6. The experimental model was highly significant (P < 0.01). The effects of both seeding line speed and seed drop height on the coefficient of variation of plant spacing were highly significant, and the effect of seeding line speed on the coefficient of variation of plant spacing was stronger than the effect of seed drop height on the coefficient of variation of plant spacing. The effect of the interaction of the two on the coefficient of variation of spacing was significant. The greater the speed of seed picking as the seed potato leaves the dorsal picking spoon, the greater the initial velocity obtained for the same drop height, resulting in a greater seed potato landing spacing. In the case of large initial velocity of seed potato, the higher the height of seed drop, creating a larger seed potato spacing at the drop point. The regression equation between the coefficient of variation of plant spacing and linear velocity of seeding line speed and seed drop height is shown in Eq (17). The regression equation was solved by planning and the coefficient of variation of plant spacing was minimized by 12.6% when the seeding line speed was 0.184 m/s and the seed drop height was 9 cm; This is mainly due to the fact that the lowest drop height reduces the speed at which seed potatoes fall to the ground. The smaller the speed, the more time the seed potato spends rolling on the back scoop while being thrown down; And there are differences between oval seed potato shapes, resulting in differences in offset distances. The greater the linear velocity of seeding, the greater the velocity of the seed potato as it comes into contact with the soil, causing greater deflection as it falls to the soil.


$$Y_{1}=27.852-111.000A-1.206B+282.222A^{2}+0.065B^{2}+0.750AB$$ (17)

(2) Significance analysis of lateral offset dispersion *Y*_2_ of seed potato

The results of the between-subjects effect test for the seed potato lateral offset dispersion *Y*_2_ is shown in Table 6. The experimental model was highly significant (P < 0.01). The effects of seeding line speed and seed drop height on seed potato lateral offset dispersion were both highly significant, with the effect of seeding line speed on seed potato lateral offset dispersion being stronger than that of seed drop height on seed potato lateral offset dispersion. The effect of the interaction of the two on the dispersion of lateral offset of seed potato was significant. The greater the picking speed, the greater the initial velocity obtained at the same seed drop height, resulting in a greater seed potato lateral offset dispersion. The interaction between the two influences the lateral offset of the seed potato landing site. The regression equation between seed potato lateral offset dispersion and seeding line speed and seed drop height is shown in Eq (18). The regression equation was solved by planning, and the seed potato lateral offset dispersion was minimized to 6.8 mm when the seeding line speed was 0.179 m/s and the seed drop height was 9.93 cm; This is mainly because the lower falling seed height reduces the speed at which the seed potatoes fall to the ground and reduces the bouncing of the seed potatoes. The smaller the seeding line speed, the more the seed potato increases the seed guide shield contact as the back seed scoop rotates around the active sprocket. The greater the velocity, the greater the uncertainty exists in the amount of bouncing displacement, causing greater deflection as the seed potato falls to the soil.


$$Y_{2}=23.122-80.944A-1.824B+205.000A^{2}+0.085B^{2}+0.750AB$$ (18)

In order to obtain the optimal operating parameters of the planter, two experimental indicators, the coefficient of variation of plant spacing and the dispersion of lateral deviation of seed potatoes, were analyzed at the minimum value; The optimal parameter combinations of the finalized factors were: the linear speed of seeding was taken as 0.184 m/s, the height of seed drop was taken as 9 cm, and the coefficient of variation of the plant spacing at this time was 12.6% and the dispersion of the lateral offset of seed potato was 6.9 mm.

#### 4.4.2 Experimental validation

After the results of the above study, it was shown that seeding line speed and seed drop height would affect the uniformity of seed potato seeding. The seed discharger apparatus worked best at a seed pickup speed of 0.184 m/s and a seed drop height of 9 cm. In order to verify the correctness of the test results, a performance comparison test was carried out on the test machine at the above parameters for three times, and the results are shown in Table 7. The test showed that the average plant spacing coefficient of variation of 12.8% and the average seed potato lateral offset dispersion of 7.0 mm were obtained, which is better than the test I did with a common single spoon on the market, further proving that the design of the double-layer picking spoon seeder was reasonable.

**Table 7:** Comparison test validation.

Project	Coefficient of variation for plant spacing (%)		Seed potato lateral offset dispersion (mm)	
	Test results	Average value	Test results	Average value
**Measured value of seed discharged from a single pick-up spoon**	18.9	18.6	13.2	12.5
	18.6		11.8	
	18.5		12.6	
**Measured value of seed discharged by double seed picking spoon**	12.4	12.8	6.5	7.0
	12.9		7.1	
	13.2		7.4	
**National standard**	≤33			

## 5. Discussion

The research object of this paper is oval potato seed potato, and the seed discharge effect is satisfactory. Compared with the existing spoon chain seed discharger, the seed discharging mechanism in this paper has two main advantages; On the one hand, in order to regulate the seeding line speed of the seed discharge chain, a tachometer is used to measure the rotational speed of the sprocket. In this way, the rotational speed of the sprocket can be precisely known for the purpose of precisely regulating it to the desired seeding line speed. On the other hand, compared with the single seed picking spoon structure, the use of double-layer spoon structure seed discharger makes the seed potato in the seed guiding area relatively fixed position, reducing the design requirements for the seed guiding shields. The difference in the initial velocity of the seed potato leaving the back picking spoon at the time of seed dropping is small, which improves the uniformity of seed potato discharging. Also, compared to the literature [24, 35], which also mentioned the test of single spoon on plant spacing coefficient of variation, the improved double pick-up spoon seed dispenser can reduce the plant spacing coefficient of variation of seed potatoes at planting. Compared with the test I have done with a common single spoon on the market, the double scoop seeder has a 5.8% lower coefficient of variation in plant spacing and a 5.5 mm lower dispersion in lateral offset.

The experiments in this paper are indoor experiments, which are somewhat different from the actual field seeding environment. The test uses a seed bed with a simulated seed discharger walking, ignoring the effect of vibration on seeding due to uneven road surface during actual seeding, the test bed provides a more ideal environment. And the lateral offset of seed potatoes during actual planting is also affected by the shape of the furrow opener.

## 6. Conclusion

1. Aiming at the potato planter in the seeding of poor uniformity of seeding problems, this paper innovatively developed a double-layer seeding spoon chain potato discharger. The force changes of seed potato in the seed picking process were analyzed, and the relevant structural parameters and working parameters were determined. The structure and parameters of the key components of the seed discharge device were studied and calculated. Structural and dimensional parameters of a double-layered seed pick-up spoon were designed to improve seed pick-up reliability and reduce the coefficient of variation of dropped seeds.
2. The trajectories of seed potatoes in the seed discharger at different seeding line speeds were analyzed by discrete element software EDEM simulation, and a proper speed was selected. Comparison verified that the single seed pickup spoon had variable drop locations and large relative positional shifts during seed pickup. And by designing the double layer seed picking spoon to make the seed potato in the seed guiding area better straightness, so as to reduce the seed potato in the seed drop position offset.
3. A test bench was set up to verify the improved seeding quality condition, with the main influencing factors of seeding speed and seed dropping height, the test bench of seed discharger was tested, and the optimal factor combination parameters were selected according to the test results. The P-value in the model is less than 0.01, indicating that the modeling in this paper is highly significant. Through the comparison test with the single pick-up seed scoop, it was verified that the seed discharge device improved the performance of seed discharge uniformity. When the seed discharging speed was 0.184 m/s and the height of seed drop was 9 cm, the coefficient of variation of plant distance was 12.8% and the dispersion of lateral offset of seed potato was 7.0 mm. In this paper, the effect of double-layer seed picking spoon on the performance of seed uniformity is better than that of the single seed picking spoon seeder, which can provide a reference for the related design of potato seeder.

## Supporting information

S1 File Experimental design and results. (XLSX)
